# Predicting the Outcomes of External Direct Current Cardioversion for Atrial Fibrillation: A Narrative Review of Current Evidence

**DOI:** 10.3390/jcdd12050168

**Published:** 2025-04-25

**Authors:** Ibrahim Antoun, Georgia R. Layton, Ahmed Abdelrazik, Mahmoud Eldesouky, Sherif Altoukhy, Mustafa Zakkar, Riyaz Somani, G. André Ng

**Affiliations:** 1 Department of Cardiology, University Hospitals of Leicester NHS Trust, Glenfield Hospital, Leicester LE5 4PW, UK; ahmed.abdelrazik@leicester.ac.uk (A.A.); mie7@leicester.ac.uk (M.E.); sherif.altoukhy@uhl-tr.nhs.uk (S.A.); riyaz.somani@uhl-tr.nhs.uk (R.S.); 2 Department of Cardiovascular Sciences, Clinical Science Wing, University of Leicester, Glenfield Hospital, Leicester LE1 7RH, UK; gl186@leicester.ac.uk (G.R.L.); mz207@leicester.ac.uk (M.Z.); 3 Leicester British Heart Foundation Centre of Research Excellence, Glenfield Hospital, Leicester LE3 9QP, UK; 4 Department of Cardiac Surgery, University Hospitals of Leicester NHS Trust, Glenfield Hospital, Leicester LE5 4PW, UK; 5 National Institute for Health Research Leicester Research Biomedical Centre, Leicester LE5 4PW, UK

**Keywords:** atrial fibrillation, direct current cardioversion, outcomes, electrocardiogram, predictors

## Abstract

Atrial fibrillation (AF) is the most common sustained arrhythmia associated with significant morbidity and mortality. External direct current cardioversion (DCCV) is a cornerstone intervention for rhythm control in AF; however, its success is influenced by various patient-specific and procedural factors. This review examines the predictors of DCCV success and AF recurrence with specific focus upon demographics, biochemical, cardiovascular imaging, and P-wave parameters and their likely ability to predict procedural outcomes. Demographic factors such as age, sex, and comorbidities influence DCCV outcomes, with prolonged AF duration, obesity, and heart failure being associated with higher failure rates. Elevated biochemical markers of inflammation and fibrosis, including C-reactive protein, galectin-3, and Type III procollagen-N-peptide, were predictive of poor outcomes. Imaging parameters, particularly left atrial (LA) volume and strain, emerged as critical indicators of atrial remodelling and DCCV failure. Increased P-wave duration and dispersion on electrocardiography were associated with an increased risk of recurrence. Biphasic waveforms and antiarrhythmic drugs, such as amiodarone and flecainide, improved cardioversion success. The predictors of DCCV success and recurrence reflect the interplay of structural, biochemical, and electrical remodelling in AF. Integrating these parameters into clinical practice can guide individualised patient management and improve outcomes. Further research is needed to validate these predictors and enhance precision medicine approaches in DCCV.

## 1. Introduction

Atrial fibrillation (AF) prevalence and incidence has grown over the last 20 years and will continue to increase over the following 30 years, becoming one of the largest public cardiovascular health challenges and epidemics globally [1,2,3,4]. External direct current cardioversion (DCCV) is a cornerstone treatment for rhythm control in patients with AF, aiming to restore sinus rhythm and improve both symptomatic and prognostic outcomes [5]. However, the success of DCCV is influenced by numerous patient-specific and procedural factors, necessitating an understanding of predictors that can optimise patient selection and post-cardioversion outcomes. It is important to note that anticoagulation is crucial before and after DCCV. The latest European Society of Cardiology guidelines emphasise the importance of delaying DCCV when the patient is not on anticoagulation and AF onset was more than 24 h earlier [5].

Demographics, imaging modalities, and electrophysiological markers have been explored as potential predictors of DCCV success and AF recurrence. Demographic factors such as age, sex, and comorbidities provide insights into underlying risk profiles, while imaging techniques like echocardiography allow for detailed cardiac structure and function assessment. Parameters derived from advanced imaging modalities, such as MRI, including left atrial (LA) size, volume, and strain, are critical markers of atrial remodelling and fibrosis and are strongly linked to AF persistence [6].

**Figure 1:**
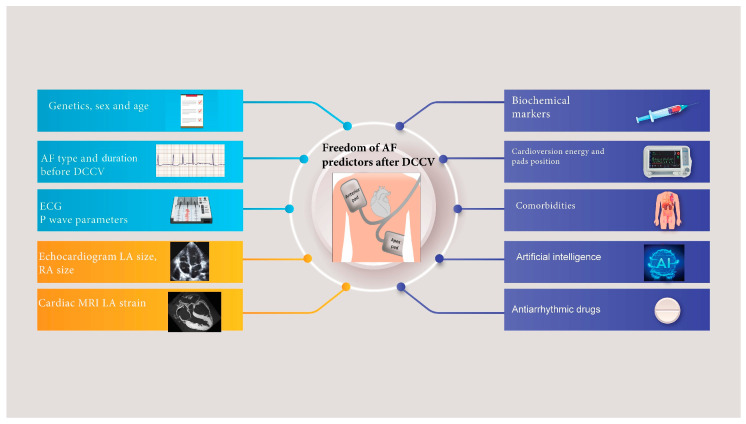
A summary of the demographic, imaging, investigative, and pharmacological factors known to predict freedom from atrial fibrillation following direct current cardioversion. DCCV: direct current cardioversion. MRI: magnetic resonance imaging. LA: left atrium. RA: right atrium. AF: atrial fibrillation.

In addition to structural and demographic factors, defibrillator pad positions, medications, and electrophysiological characteristics, particularly those related to the P-wave [7], offer a unique perspective on atrial conduction and arrhythmogenic potential. P-wave duration, dispersion, and amplitude reflect atrial electrical remodelling and may be non-invasive markers for predicting cardioversion success and AF recurrence risk. This review examines the current evidence on predictors of DCCV success and recurrence in AF, focusing on demographic characteristics, procedural aspects, cardiovascular imaging, and P-wave metrics. By synthesising findings across these domains, this review seeks to identify gaps in knowledge, highlight areas for future research, and support clinical decision-making for individualised management strategies. This review adopts a narrative approach to synthesise a summary review of existing evidence regarding the broad predictors of outcomes following external direct current DCCV for AF (Figure 1). Whilst a systematic review methodology, as outlined by PRISMA guidelines, provides structured appraisal, our objective was to integrate the diverse types of evidence, including demographic, biochemical, cardiovascular imaging, and electrophysiological predictors, into a cohesive summary. This review aims to highlight interdisciplinary connections, emphasise clinical context, and identify current gaps that systematic methods might not adequately capture.

### 1.1. Technical Predictors

**Figure 2:**
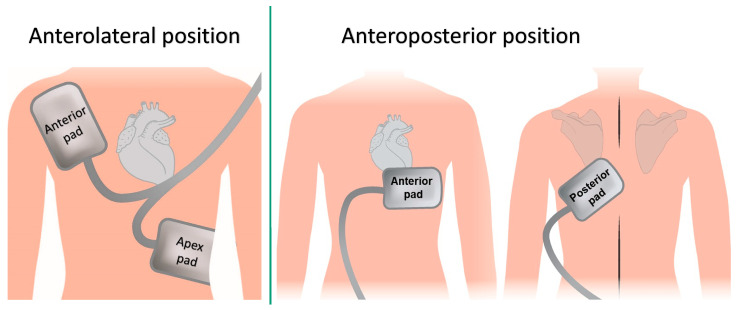
Anterolateral and anteroposterior external cardioversion positions.

The positioning of defibrillator pads is a critical factor influencing the success rates of electrical cardioversion in patients with AF. Various studies have explored the optimal pad placement and its implications for cardioversion outcomes (Figure 2). Notably, the anterior–lateral configuration was suggested to be superior to the anterior–posterior configuration for successful cardioversion, according to two studies [8,9]. Another study indicated that the anterior–posterior position was superior to the anterolateral for persistent AF [10]. Two later studies have shown that the exact location of the pads was not crucial in determining DCCV outcome [11,12]. In addition to pad positioning, the type of waveform used during cardioversion also plays a significant role. A systematic review comparing biphasic and monophasic waveforms found that biphasic waveforms are generally more effective in achieving successful cardioversion outcomes [13]. The increasing number of previous cardioversions independently predicts the risk of AF recurrence following DCCV, with a higher risk of early recurrence associated with a greater number of prior cardioversions [14].

Furthermore, total energy delivered during the procedure is crucial. A single-centre study proposed that three shocks with 360J were more effective than escalated shocks at 125-150-200 J [15]. Manual pressure on the pads has also increased success rate, especially in obese patients [16]. Additionally, the type of electrodes used in cardioversion is important. In a randomised clinical trial involving 201 patients, hand-held paddles improved the odds of DCCV’s success compared to the adhesive patches [17], especially in obese patients [18]. The same study proposed that manual pressure augmentation techniques can benefit these patients [18].

### 1.2. Antiarrhythmic Drugs Use

The use of antiarrhythmic drugs (AADs) following DCCV increased the success rate [19,20] with several agents have been suggested to improve sinus rhythm (SR) maintenance:

1. Flecainide and Propafenone: Both medications are classified as class IC antiarrhythmics and are known for their efficacy in converting AF to sinus rhythm, particularly in patients with paroxysmal AF. The effectiveness of these agents is comparable, with studies indicating that both flecainide and propafenone can achieve similar conversion rates to SR following DCCV [21]. Furthermore, flecainide is often recommended in clinical guidelines for early pharmacologic cardioversion due to its rapid onset of action and favourable safety profile in selected patients [22]. In addition to their efficacy in restoring sinus rhythm, flecainide and propafenone may influence long-term outcomes following DCCV. Research indicates that early rhythm control strategies, including administering these antiarrhythmics, can reduce the risk of AF recurrence and improve overall cardiovascular outcomes [23]. For instance, a systematic review highlighted that early pharmacologic cardioversion with agents like flecainide not only facilitates immediate conversion but may also serve as a bridge to longer-term rhythm control strategies [23,24].

Moreover, integrating these medications into managing AF can help mitigate the risk of thromboembolic events associated with cardioversion, particularly in patients with a high CHA_2_DS_2_-VA score [25]. However, it is crucial to consider the safety profile of these agents. Both flecainide and propafenone carry a risk of arrhythmia, particularly in patients with structural heart disease or significant left ventricular dysfunction [21,25]. Therefore, careful patient selection and monitoring are essential to optimise the benefits of these medications while minimising potential adverse effects. The decision to use flecainide or propafenone should be guided by individual patient characteristics, including the duration of AF, underlying heart conditions, and previous responses to antiarrhythmic therapy [23,26].

2. Amiodarone is a widely utilised antiarrhythmic medication particularly in AF, where it is often administered to improve outcomes following DCCV. The efficacy of amiodarone in facilitating successful conversion from AF to SR has been documented in various studies, highlighting its role as a first-line agent in this setting. Research indicates that amiodarone can significantly enhance the success rate of electrical cardioversion according to a meta-analysis [27]. In addition to its efficacy, amiodarone’s pharmacological properties contribute to its effectiveness in this context. It acts by blocking sodium, calcium, and potassium channels and exhibiting beta-blocking effects, which help stabilise cardiac rhythm and facilitate successful cardioversion [28]. Combining electrical cardioversion with amiodarone administration has been shown to reduce the risk of immediate recurrence of AF following the procedure, thereby improving overall patient outcomes [29]. However, the use of amiodarone is not without concerns. While it is effective, there are potential risks associated with its administration, including the development of torsades de pointes, particularly in patients with underlying heart conditions [30]. Moreover, some studies have indicated that the pre-treatment with amiodarone does not always yield statistically significant improvements in cardioversion success rates compared to those who do not receive the drug, suggesting that its benefits may vary among different patient populations [31].
3. Sotalol: Another class III antiarrhythmic agent commonly used in AF care pre-DCCV. In a systematic review and meta-analysis, sotalol demonstrated significant efficacy in the pharmacologic conversion of AF, with a higher rate of successful cardioversion than placebo [32]. Specifically, one study reported that sotalol had a cardioversion rate of 68% in rhythm control groups, significantly higher than the 42% observed in rate control groups [33]. This highlights Sotalol’s potential as a first-line agent for rhythm control in patients undergoing DCCV. Furthermore, intravenous sotalol has been explored as a rapid loading strategy, which may enhance the speed of achieving therapeutic levels and improve patient outcomes [34,35]. However, the safety profile of sotalol must be considered, particularly regarding its association with QT interval prolongation and the risk of TdP [36,37]. The American Heart Association (AHA) guidelines recommend careful monitoring of patients receiving sotalol, especially those with pre-existing conditions such as heart failure or renal impairment [38]. Despite these concerns, when used appropriately, sotalol has shown to be a safe and effective option for maintaining SR after DCCV, with studies indicating that the risk of adverse effects can be managed through careful patient selection and monitoring protocols [33,36].

### 1.3. Patient Factors (Demographics and Clinical Parameters)

Several patient demographics (including age, gender, and functional capacity) and clinical factors are associated with higher rates of AF recurrence after DCCV (Table 1) [39]. Older age, generally due to more structural atrial remodelling, maintain sinus rhythm at lower rates than younger patients [40]. Similarly, prolonged AF duration prior to DCCV is a significant predictor of failure due to the structural and electrical remodelling of the atria [41]. This is exacerbated by obesity through atrial enlargement, increased inflammation, and elevated left atrial pressure [41,42]. Left atrial size often signifies more several atrial remodelling or conduction disturbance, resulting in a higher likelihood of atrial recurrence [6]. Therefore, it is unsurprising that the presence of structural heart diseases which are also associated with left atrial enlargement, such as heart failure and valvular disease, also demonstrate higher risks of recurrence [6]. Female gender is associated with a higher risk of failure, potentially due to differences in atrial size as well as the hormonal influences of post-menopause [43]. At the same time, ischemic heart disease contributes to atrial fibrosis and scarring, reducing the success of cardioversion [44]. Advancing age further impairs outcomes due to age-related atrial fibrosis and reduced compliance [45], and heart failure—whether with reduced or preserved ejection fraction—worsens atrial remodelling and electrical dysfunction, making successful rhythm restoration more challenging [46,47,48]. Comorbid conditions such as diabetes mellitus [49], obstructive sleep apnoea (OSA) [50], renal impairment [51], and chronic obstructive pulmonary disease (COPD) also contribute to poor outcomes [52]; diabetes promotes atrial stiffness and inflammation, OSA induces atrial stretch and heightened sympathetic activity, renal impairment leads to systemic inflammation and volume overload, and COPD exacerbates arrhythmogenesis through hypoxia and right ventricular strain. The underlying pathology of AF influences the outcomes of DCCV. Most patients with hyperthyroidism-induced AF spontaneously restore their sinus rhythm following the return of the euthyroid state [53]. However, some patients develop hyperthyroidism-induced persistent AF, and this group has a significantly higher risk of recurrence following cardioversion compared to persistent AF of other origins [54]. Psychological factors like depression and anxiety further impair cardioversion success by disrupting autonomic balance and adherence to therapy [55]. At the same time, a high frailty score reflects reduced physiological reserve and a higher burden of comorbidities, which negatively affect procedural outcomes [56]. These factors highlight the multifaceted nature of DCCV failure and underscore the importance of individualised patient assessment and management.

### 1.4. Biochemical Markers

Several biochemical markers have been identified as predictors of DCCV outcomes in AF, reflecting the roles of inflammation, fibrosis, and atrial remodelling (Table 2). Elevated inflammatory markers, such as C-reactive protein (CRP), have been linked to poor DCCV success [59]. Fibrotic markers play a key role, including increased Type III procollagen-N-peptide [60], galectin-3 [61], and reduced circulating endothelial progenitor cells, which indicate endothelial dysfunction [62]. Elevated cardiac stress markers, such as B-type natriuretic peptide (BNP), correlate with atrial stretch and poor outcomes [63]. Additionally, increased interleukin-2 and heat shock protein 70, involved in inflammatory and cellular stress responses, have been linked to lower success rates [64]. Electrolyte disturbances like hypokalaemia and hypomagnesemia increase the occurrence of AF [65,66]. Therefore, correcting these imbalances before the procedure is crucial to optimising DCCV outcomes. These findings emphasise the multifactorial nature of DCCV outcomes, integrating inflammatory, fibrotic, genetic, and cardiac stress factors.

### 1.5. Genetic Predisposition

Genetic variants are increasingly recognised as potential contributors to AF susceptibility and recurrence, with emerging data suggesting they may also influence the efficacy of DCCV. Although the clinical utility of genetic screening remains limited, several polymorphisms—particularly on chromosome 4q25 near the *PITX2* gene—have been associated with a higher likelihood of AF recurrence following successful DCCV [63,64]. These variants are believed to affect atrial electrical properties and structural remodelling, thereby lowering the threshold for AF reinitiation after cardioversion.

In addition to 4q25, mutations in genes encoding cardiac ion channels—such as *SCN5A*, *KCNQ1*, and *CACNA1C*—modulate sodium, potassium, and calcium currents, respectively, altering atrial conduction and excitability [65,66]. Variants in structural genes like *NUP155* and *LMNA*, previously implicated in familial AF, may also play a role in atrial myopathy, thereby influencing cardioversion outcomes [67]. While most studies have focused on AF pathogenesis broadly, the relationship between these variants and DCCV outcomes specifically is gaining traction. For example, Shoemaker et al. [64] demonstrated that patients carrying the *PITX2*-linked 4q25 risk allele had significantly higher AF recurrence rates post-DCCV, independent of clinical risk factors.

Efforts to integrate genetic markers into risk prediction models have shown modest improvements in prognostic accuracy [68]. However, given that known variants account for only a small proportion of AF heritability, their standalone predictive value for DCCV outcomes remains limited. The development of polygenic risk scores and their combination with clinical and electrophysiological parameters may offer a more robust predictive framework in the future. Further research is needed to validate these approaches across diverse populations and to explore gene–environment interactions that modulate procedural success.

### 1.6. Cardiovascular Imaging

Cardiovascular imaging has proven to be a valuable tool for assessing structural and functional characteristics, whether echocardiography or magnetic resonance is used. It aids in predicting the success of DCCV in AF. Several imaging parameters correlate with DCCV outcomes, providing insight into the underlying atrial substrate and its impact on rhythm restoration.

Two studies have demonstrated that reduced left atrial (LA) appendage flow velocity is a key predictor of DCCV failure [69,70]. Decreased appendage flow velocity indicates impaired atrial contractility and may reflect advanced atrial remodelling, which reduces the likelihood of maintaining sinus rhythm. Atrial strain reflects the atrial myocardium’s ability to deform during the cardiac cycle, and its reduction signifies decreased atrial compliance and fibrosis.

Structural atrial remodelling also plays a significant role in DCCV success. Two studies by Marchese et al. demonstrated that an increased left atrial volume index (LAVI), an indicator of atrial enlargement, correlates with a higher likelihood of DCCV failure [71,72]. Additionally, valvular AF, particularly in patients with mitral valve disease, is strongly associated with increased LA volume [73], which, according to a recent meta-analysis, impairs the outcomes of DCCV and increases the recurrence rates [6].

While LAVI is significant, an increase in right atrial (RA) volume exceeding LAVI is an even stronger predictor of poor outcomes [74], emphasising the importance of evaluating both atria in predicting rhythm control success. Similarly, two studies confirmed that left atrial dilation is strongly associated with a reduced probability of successful cardioversion [75,76]. Atrial dilation reflects chronic pressure overload, structural remodelling, and fibrosis, all of which impair atrial electrical function (Table 3).

### 1.7. Electrocardiogram Markers

The assessment of AF outcomes following DCCV can be significantly enhanced by utilising various electrocardiographic (ECG) predictors. A comprehensive understanding of these predictors is essential for optimising patient management and improving clinical outcomes. The main predictive ECG parameters described are P wave-related. Increased P wave duration (PWD) and dispersion have been associated with DCCV failure [7,78,79,80,81,82,83,84,85,86,87,88,89,90,91,92] (Table 4). It is evidence that integrating multiple ECG predictors, mainly P-wave dispersion and PWD, can significantly enhance the prediction of DCCV outcomes in AF patients [93].

## 2. The Role of Artificial Intelligence

Emerging evidence highlights the promising role of machine learning algorithms and artificial intelligence (AI) in predicting outcomes after electrical cardioversion for AF. These advanced computational approaches can effectively analyse and integrate large-scale heterogeneous datasets, encompassing clinical demographics, biochemical markers, cardiovascular imaging, and electrophysiological parameters, into robust predictive models. Recently, machine learning models demonstrated superior predictive performance compared to traditional statistical methods, providing more personalised and accurate risk stratification. Specifically, Kwon et al. developed a predictive model using machine learning algorithms, accurately forecasting AF recurrence after electrical cardioversion among patients with persistent AF. Their findings illustrate how integrating diverse clinical data through advanced machine-learning techniques significantly enhances prognostic precision [94]. Additionally, Núñez-García et al. established and validated a prognostic model leveraging machine learning to predict cardioversion outcomes, further underscoring the potential of these methods to refine patient selection, optimise clinical decision-making, and improve patient-specific outcomes following cardioversion [95]. Nevertheless, despite these promising developments, further validation in larger, multicentre cohorts and prospective studies is essential to ensure generalizability and effective integration of AI-derived models into routine clinical practice. Future research directions should also address AI models’ interpretability and real-world applicability within clinical workflows, thereby enabling widespread adoption in personalised AF management strategies.

## 3. Conclusions

This review article analyses all well-established predictors of DCCV outcomes. A comprehensive understanding of these predictors is essential for optimising patient selection, improving procedural success, and reducing AF recurrence. This review highlights the multifactorial nature of DCCV outcomes; integrating evidence from demographic, biochemical, imaging, and electrocardiographic domains. Demographic factors such as age, sex, and comorbid conditions, combined with structural changes identified through advanced imaging modalities, underscore the significant influence of atrial remodelling on cardioversion success. Biochemical markers provide critical insights into the roles of inflammation, fibrosis, and atrial stress in AF persistence, while genetic polymorphisms and electrophysiological characteristics, particularly P-wave metrics, offer additional tools for risk stratification. Cardiovascular imaging, particularly echocardiography, provides crucial insights into atrial structure and function, with parameters such as left atrial volume index, atrial strain, and appendage flow velocity offering prognostic value. Right atrial enlargement and biatrial remodelling further enhance risk stratification. These imaging-derived markers complement demographic and biochemical predictors to inform clinical decision-making. 

Technical aspects of DCCV, including pad positioning, waveform type, and the use of antiarrhythmic drugs, also play pivotal roles in improving procedural outcomes. Advances in cardiovascular imaging and electrophysiology have further enhanced the ability to predict success and guide individualised treatment strategies.

Despite these advances, the current literature reveals significant gaps in knowledge. Many predictors lack consensus or require further validation through large, prospective, and multicentre studies. Future research should also explore integrating these predictors into practical clinical decision-making algorithms, focusing on precision medicine approaches that tailor interventions to individual patient profiles. We acknowledge the inherent limitations of narrative reviews, including potential selection bias and less reproducibility compared to systematic reviews. Nonetheless, our integrative narrative approach highlights the multifaceted interactions influencing cardioversion outcomes and provides clinically relevant insights that can inform future systematic evaluations. In conclusion, whilst our understanding DCCV predictors has significantly improved with time, continued interdisciplinary research is essential to refine this knowledge and to use it to generate predictive tools and integrate these effectively into clinical practice. By doing so, the management of AF in patients undergoing DCCV can be better optimised, improving both early and late outcomes and minimising the long-term morbidity of this condition.

## Figures and Tables

**Table 1:** The role of demographics and clinical factors in predicting electrical cardioversion outcomes for atrial fibrillation.

Study	Study Identified Demographic or Clinical Factor Associayed with DCCV Failure
Frick et al., 2001, Brodsky et al., 1989 [41,42]	Obesity, Increasing AF duration
Suttorp et al., 1993 [44]	Females, Ischaemic heart disease
Alt et al., 1997 [45]	Increasing age
van den Berg et al., 1998, Caputo et al., 2011, Melduni and Cullen, 2012 [46,47,48]	Heart failure (reduced and preserved ejection fraction)
Lange and Herrmann-Lingen, 2007 [57]	Low mood
Soran et al., 2008 [49]	Diabetes melitus
Kanagala et al., 2003 [50]	Obstructive sleep apnoea
Schmidt et al., 2011 [51]	Renal impairment
Pisters et al., 2012 [52]	Chronic obstructive lung disease
Mlynarska et al., 2020 [56]	High frailty score
García-Izquierdo et al., 2020 [58]	Anxiety

**Table 2:** Biochemical predictors of direct current cardioversion failure for atrial fibrillation.

Study	Biochemical Markers Relation to Direct Current Cardioversion Failure
Liu et al., 2007 [59]	Increased C-reactive protein
Siu et al., 2009 [62]	Circulating endothelial progenitor cell count ↓ (fibrotic marker)
Kawamura et al., 2012 [60]	Type III procollagen-N-peptide ↑ (fibrotic marker)
Parvez et al., 2013, Shoemaker et al., 2015 [67,68]	Polymorphisms on the 4q25 chromosome
Zografos et al., 2014 [63]	Increased B-type natriuretic peptide
Gürses et al., 2019 [61]	Galectin 3 (fibrotic marker)
Rigopoulos et al., 2021 [64]	Serum interleukin 2 ↑, heat shock protein 70 ↑ (involved in cellular protein folding)

↑: increase. ↓: decrease.

**Table 3:** Correlation between cardiovascular imaging and cardioversion recurrence.

Study	Imaging Correlation with DCCV Failure
Verhorst et al., 1997, Kostakou et al., 2021 [69,70]	LA appendage flow ↓
Di Salvo et al., 2005 [77]	Atrial strain ↓
Marchese et al., 2010, Marchese et al., 2012 [71,72]	LAVI ↑
Luong et al., 2015 [74]	RA volume ↑ > LAVI ↑
Fornengo et al., 2015, Toufan et al., 2017 [75,76]	LA dilation
Marques-Alves et al., 2020 [73]	Mitral valve disease

↑: increase. ↓: decrease.

**Table 4:** Correlation between P-wave parameters and DCCV recurrence.

Author and Year	AF	n	Follow-up	ECG	Parameter	Recurrence Change	Recurrence Cut-off
Opolski et al., 1997 [78]	PersAF	35	6 months	SAECG	PWD	↑	>137 ms
Stafford et al., 1998 [81]	PersAF (77%)	31	1 week	SAECG	P wave energy	↑	25%> drop
Aytemir et al., 1999 [82]	PersAF	73	6 months	SAECG	Filtered PWD	↑	>128 ms
Raitt et al., 2000 [83]	PersAF	20	1 year	SAECG	PWD	↑	>130-140 ms
Guo et al., 2003 [85]	PersAF	60	6 months	SAECG	Filtered PWD	↑	Nil
Ehrlich et al., 2003 [86]	No mention	111	1 week	SAECG	PWD	↑	>145 ms
Dixen et al., 2004 [84]	PersAF	131	1 month	SAECG	PWD	↑	>160 ms
Dogan et al., 2004 [80]	PersAF (45%)	64	6 months	SAECG	PWDisp	↑	>46 ms
Perzanowski et al., 2005 [87]	PersAF	45	6 months	SAECG	PWDisp	↑	>80 ms
Budeus et al., 2005 [88]	PersAF	141	1 year	SAECG	PWD	↑	>126 ms
Başar et al., 2011 [89]	PersAF	26	1 year	12 leads	PWDisp	↑	
Gonna et al., 2014 [90]	PersAF	77	1 month	12 leads	PWD	↑	>125 ms
Blanche et al., 2014 [91]	PersAF	133	9 months	SAECG	Nil	Nil	Nil
Fujimoto et al., 2018 [92]	PersAF	141	1 month	12 leads	PWDisp	↑	Nil
Choi et al., 2021 [79]	PersAF	272	2 months	12 leads	PWD, PTFV1	↑	>134 ms, >50 ms.mm
Antoun et al., 2024 [7]	PersAF	52	12 months	Body surface mapping	PWD	↑	>161 ms

PersAF: persistent atrial fibrillation. PWD: p wave duration. PWDisp: p wave dispersion. PTFV1: p wave terminal force in V1. SAECG: signal averaged electrocardiogram. Nil: nothing. ↑: increase. ↓: decrease.

## Data Availability

Data relating to this study are available upon reasonable request from the corresponding author.
